# Ejaculate Characteristics Depend on Social Environment in the Horse (*Equus caballus*)

**DOI:** 10.1371/journal.pone.0143185

**Published:** 2015-11-24

**Authors:** Dominik Burger, Guillaume Dolivo, Claus Wedekind

**Affiliations:** 1 Swiss Institute of Equine Medicine, Agroscope and University of Berne, Avenches, Switzerland; 2 Department of Ecology and Evolution, Biophore, University of Lausanne, Lausanne, Switzerland; University of Melbourne, AUSTRALIA

## Abstract

Sperm competition theory predicts semen characteristics to be affected by the social environment. We used the polygamous horse (*Equus caballus*) to experimentally study within-subject plasticity in response to different social environments. Stallions were sequentially exposed, over a period of 8 weeks each, to other stallions and then singly to mares, or vice versa (in adjacent boxes separated by grills). Ejaculates were collected to determine semen characteristics. Highest sperm numbers were found in stallions that were first exposed to other stallions and then to mares, while lowest sperm numbers were observed in stallions that had been exposed to mares but not yet to other stallions. One of three sperm velocity measures (curvilinear velocity) was consistently elevated in stallions that were first exposed to stallions and then to mares. Sperm number after exposure to mares and curvilinear sperm velocity after exposure to stallions were both positively correlated to average blood testosterone levels during the corresponding period of exposure. We conclude that ejaculate characteristics are plastic traits affected by the social environment in horses.

## Introduction

Selection is predicted to affect the evolution of ejaculate characteristics in species where females have the possibility to mate multiply and hence sperm of different males may have to compete for fertilization of eggs [1,2]. Key ejaculate characteristics that are predicted to be shaped by this evolution are (i) sperm number per ejaculate [3] and (ii) sperm velocity [4,5]. The latter is affected by sperm morphology and ATP content [5,6]. Sperm velocity has been shown to be an important predictor of fertilization success in some fish [7].

Because sperm production is costly [8], optimal ejaculate expenditure may not only evolve in response to the degree of sperm competition but may also display plasticity in response to changes in mating roles or temporal changes in sperm competition [9]. Such plasticity has been intensely studied in invertebrates [3] and has been found at the level of sperm production [10,11] and at the level of sperm allocation (i.e. the investment into a particular mating; recent studies include Arudell et al. [12], and Kimura and Chiba [13]). Sperm number may be the trait that is most likely to be phenotypically plastic, as suggested by Kelly and Jennions [3] in their recent meta-analysis. However, experiments in fish demonstrate that sperm velocity can sometimes be quickly adjusted in response to male-male interactions [14,15] or in response to the presence of females [16].

Within mammals, studies on the evolution and plasticity of ejaculates have concentrated on rodents. Comparisons within rodents found species differences in mean ejaculate quality and sperm characteristics to be correlated to body size, energy budget, and the average level of sperm competition [17,18]. Within-species studies on rodents suggest plasticity in response to the risk of sperm competition in sperm production and sperm allocation [11,19–23]. Less is known about such within-subject plasticity in other mammalian taxa with different life histories.

We choose the polygamous horse (*Equus caballus*) as experimental model to test whether semen characteristics such as sperm number and sperm velocity can be influenced by the social environments stallions are exposed to. Under feral conditions, mares live throughout the year in fairly stable social and breeding bands called harems [24]. Harems usually include one and sometimes up to five mature stallions, typically with several mares with or without offspring, but a “harem” size of just one mare is possible [25,26]. Non-harem stallions will often form so-called “bachelor stallion bands” with fluctuating group size. Bachelors sometimes have the opportunity to mate with mares previously dispersed from a harem band.

Stallions typically show much behavioral plasticity in response to different social environments [24], and analogous plasticity in semen parameters has been hypothesized [27]. We therefore compared two environments that are likely to create extreme differences in predicted optimal semen investments: exposure to a group of other stallions versus exposure to one mare only. Each stallion was exposed to both situations in order to allow for statistically powerful within-subject comparisons, and exposure to each type of environment lasted over a period of eight weeks, with determination of ejaculate characteristics in the ninth week, to cover the 57 days of spermatogenesis [28].

Testosterone is a steroid hormone that has numerous functions in various contexts, including the regulation of time and energy put into competitive and sexual behavior such as mate seeking, male-male competition, dominance, mating effort, paternal behavior, and the degree of polygyny [29,30], with testosterone secretion typically showing high inter- and within-species plasticity [31]. Several studies in mice and other mammals have found that exposure of males to females or their scent induces a “testosterone surge” that activates a variety of courtship and mating behaviors [32–34], see Khalil et al. [35] for a recent example in horses. Wingfield et al. [36] predicted that patterns of testosterone secretion are useful proxies of male reproductive strategies (see also [37]). We therefore also tested whether peripheral blood testosterone levels correlate with semen characteristics under the two extreme social environments we created.

## Methods

### Exposure to stallions and mares

Twelve breeding stallions (Franches-Montagnes horses, with stud records showing normal fertility) and six mares (three Franches-Montagnes and three Warmblood horses) were used. Previously all stallions had been stabled in individual stud housing systems. For the experiment they were kept in seven experimental stables of two types: six identical stables containing four boxes in two pairs (12 m^2^ per box) and a 2.90 m wide corridor in between, with solid wood up to a height of 1.3 m and a metal grille above as separation between the boxes and towards the corridor. This allowed visual, olfactory, and limited tactile contact between the animals. The seventh stable consisted of two rows of four boxes but was otherwise identical to the others. All boxes were bedded with straw.

The experiment was carried out in a crossed design during two time periods. In period one (starting on Monday Aug 8^th^, 2011), six stallions (randomly chosen among the 12 stallions) were distributed to six of the eight boxes of the large stable for a period of eight weeks (group “stallion contact”). The other six stallions were housed together with one mare in one of the six separate stables each, with the stallion and the mare in adjacent boxes (group “mare contact”; the remaining two boxes per stable remained empty). Four weeks later, all mares were newly assigned to the stallions in the course of a parallel study on male responses to female signals [38], i.e. each stallion was sequentially exposed to two different mares during the eight weeks in the “mare contact” group (this additional treatment within the “mare contact” group was full-factorial and fully balanced, i.e. it could not confound any conclusions drawn from the present study). After a transitory week during which ejaculates were collected (see below), all 12 stallions were switched from one group type to the other and the experiments were repeated (period two, starting Oct 10^th^, 2011) so that, by the end of the study, all stallions had been in all possible experimental groups and the experimental design was fully balanced (see Janett et al. [39] for a discussion of seasonality of sperm parameters of Franches-Montagnes stallions). All six mares turned out to be cycling normally throughout the experiment. They were all at least once (range 1–2) in estrus during each exposure to a different stallion.

All experiments were performed in the Swiss National Stud Farm. During the experimental period, there were in total 59 stallions, three geldings, and 23 mares on the farm, the mares being spatially separated from the males. The six experimental “mare contact” stables were located at the periphery of the stud in order to keep the possibility of olfactory, auditory, and visual communication to non-experimental animals low. The “stallion contact” stable was located in the center of the stud, i.e. the possibility of any type of communication to non-experimental animals was higher for this experimental group.

### Testosterone blood levels and semen characteristics

During the entire experiment, blood samples (EDTA) were collected from the stallions once per week for testosterone analysis. This was done every Wednesday between 10 and 10.30 a.m. via jugular venipuncture. The samples were immediately centrifuged (4,000 x g for 10 min) and the plasma frozen (-80° C for less than three months) until analysis. Testosterone was determined via electrochemiluminescence immunoassay (Elecsys 2010, Roche Diagnostics, Basel, Switzerland) as described and validated in Janett et al. [40] (inter- and intra-assay coefficients of variation were 2.2 and 1.4%, respectively).

During the week that followed each eight weeks block of exposure to either stallions or mares, stallions were kept in the same situation (stallion or mare contact) and semen was collected three times (Monday, Wednesday, and Friday) in order to determine average sperm number and average sperm velocity in response to the treatment. Semen collection was performed in a separate room using an artificial vagina (Avenches model, Switzerland) and a phantom with always the same ovariectomized stimulus mare standing in a box in front of the phantom, i.e. following a standard procedure that all stallions had frequently experienced before. This stimulus mare had not been included in the other treatments. Extra-gonadal sperm reserves [41,42] were minimized in daily semen collections (Monday–Friday) the week before each, i.e. during the eighth week of each experimental period, to deplete potentially accumulated semen reserves in the cauda epididymidis and to allow for accurate determination of daily sperm output and other semen characteristics [43,44] (e.g. to correct for the potentially confounding effects of sperm resorption in the epididymides or loss in the urine [41]).

Immediately after semen collection, total sperm number was calculated from ejaculate volume and sperm concentration as determined in a nucleocounter (SP-100, ChemoMetec, Allerød, Denmark). Sperm velocity was assessed in 10 μl raw semen diluted in 20 μl INRA 96^TM^ (IMV, L’Aîgle, France) and with a computer-assisted sperm analyzer (HTM-IVOS, Version 12, Beverly, MA, USA) using a 20 μm standard count analysis chamber (Art. Nr. SC 20-01-C, Leja, Nieuw-Vennep, Netherlands) set at 37.5°C. Three measures of sperm velocity were taken (all in μm/s): the straight line velocity (VSL) deduced from the average distance between the sperm heads’ first detected positions to their last, the curvilinear velocity (VCL) deduced from an approximation of the curvilinear path the sperm heads took, and the average path velocity (VAP) deduced from the (smoothed) paths the sperm heads took during the observational period.

### Statistics and ethical note

Treatment effects on testosterone blood levels (means over eight weeks each) and semen characteristics (means over three semen samples taken each in the week following an eight-week period) were tested as repeated measures in MANOVAs (after graphical inspection of the data suggested that the model assumptions were not significantly violated), with the order of exposing stallions to other stallions or to mares included as fixed factors to account for potential order effects. Kendall rank correlation coefficients (tau) were used to analyze correlations, because in some cases the model assumptions for parametric correlation analyses seemed not sufficiently fulfilled.

Animal experimentation was performed following approval from the local animal ethics committee (Etat de Vaud, Service Vétérinaire, approval #2454). The animals had ad libitum access to water and were fed three times per day with hay, oats, barley, corn, and pellets supplemented with minerals. The stallions were regularly and individually exercised and had daily access for about one hour to a paddock without any direct contact to other stallions or mares. Mares were turned out daily in groups for three hours in paddocks without any stallion contact. All horses had been dewormed before the experiments, seemed healthy, and absence of intestinal parasites could be confirmed using the McMaster method with a detection limit of 50 EpG [45] on feces samples.

Towards the end of the experiment, a 20-year old stallion who had already gone through the “mare contact” treatment had to be euthanized in the third week of the “stallion contact” treatment due to a sudden and unexplained rupture of its urinary bladder. This stallion appeared healthy during all exposures to mares and during the first two weeks in the stallion group. Therefore, all available measurements were included in the analyses, i.e. his mean testosterone value for the “stallion contact” treatment was based on the first two weekly measurements within this treatment.

## Results

**Fig 1:**
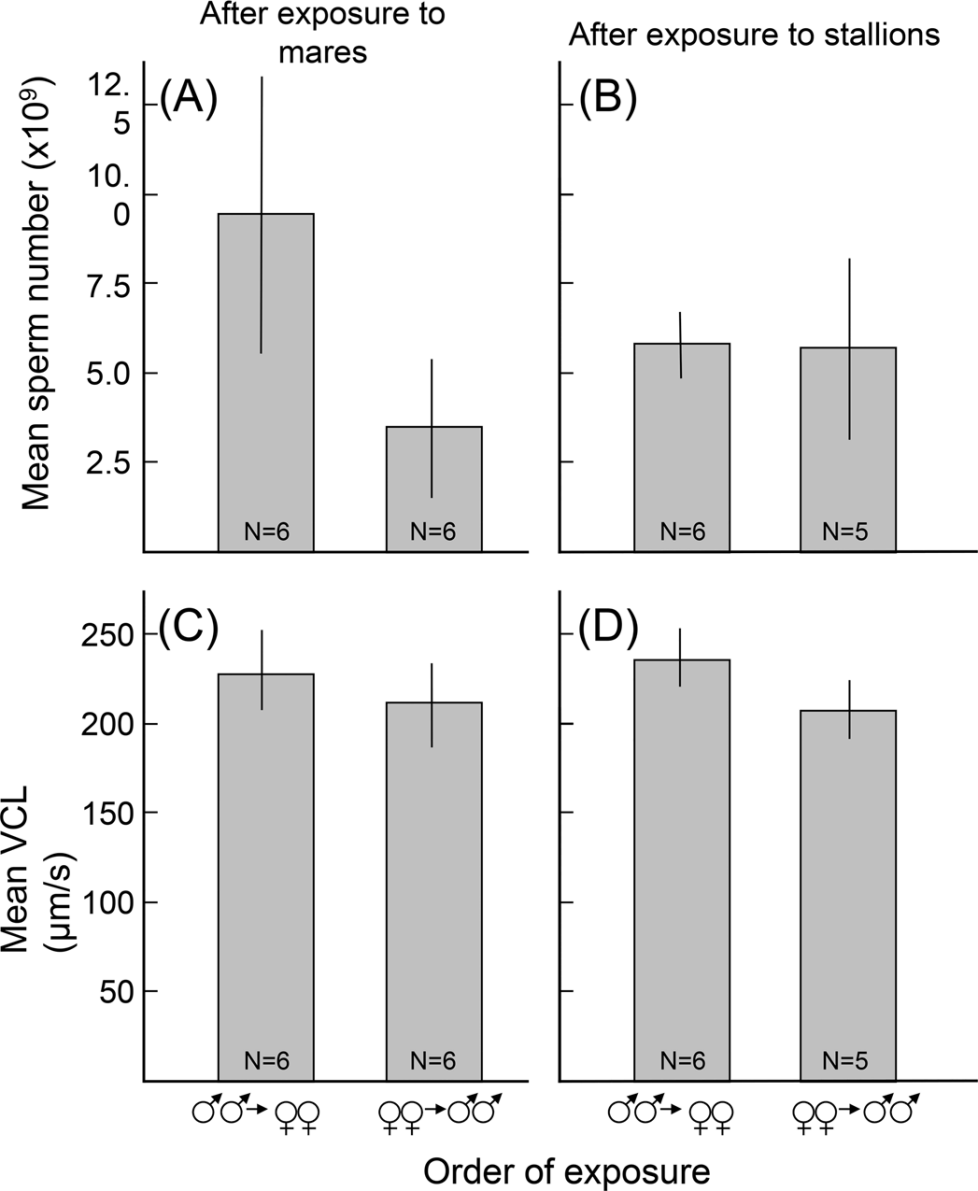
Semen characteristics. Semen characteristics (means ± 95% CI over averages of three ejaculates per stallion) after exposure to mares (A, C) or to stallions (B, D), plotted separately for mean sperm number (A, B), mean VCL (C, D), and each for stallions who were first exposed to other stallions and then to mares, and for stallions who were first exposed to mares and then to other stallions. The numbers of stallions per treatment are indicated in the figure (one stallion had to be euthanized before the last semen collection–see Methods). See Tables 1 and 2 for statistics.

The order of exposure to stallions and mares significantly affected ejaculate characteristics: Sperm numbers were highest after exposure to mares for stallions that had previously been exposed to other stallions, and lowest after exposure to mares for stallions that had not previously been exposed to other stallions (Fig 1A; see interaction term in Table 1), while sperm numbers were about in between after exposure to stallions, regardless of the order of exposure (Fig 1B). When controlling for these order effects, sperm numbers did not seem to be affected by the exposure to other stallions or to mares only (within-subject main effect in Table 1).

**Table 1:** MANOVA on mean sperm number after exposure to stallions or to mares.

	d.f.	F	p
*Between-subjects*:			
Order of presentation (stallions or mares first)	1, 9	5.50	0.04
*Within-subjects*:			
Effect of stallions or mares	1, 9	1.65	0.23
Effect of stallions or mares x order	1, 9	12.2	0.007

Sperm numbers were never significantly correlated to the corresponding sperm velocity measures (|tau| always < 0.30, p always > 0.17). Among the measures of sperm velocity, VCL was consistently higher in stallions that had been exposed to other stallions first than in those that had been exposed to mares first (Table 2; Fig 1C and 1D). Although corresponding velocity measures were mostly correlated (five of six possible pairwise correlations were significant; range: 0.24 < tau < 0.69), no such treatment effects could be seen in the other measures of sperm velocity (Table 2). Moreover, no measure of sperm velocity seemed to be affected by the exposure to other stallions or to mares when controlling for possible order effects (Table 2).

**Table 2:** MANOVA on the mean sperm velocity measures VSL (straight line velocity), VCL (curvilinear velocity), and VAP (average path velocity) after exposure to stallions or to mares.

		VSL		VCL		VAP	
	d.f.	F	p	F	p	F	p
*Between-subjects*:							
Order of presentation	1, 9	0.84	0.38	5.94	0.04	3.89	0.08
*Within-subjects*:							
Effect of stallions or mares	1, 9	0.90	0.37	0.47	0.51	0.03	0.87
Effect of stallions or mares x order	1, 9	0.97	0.35	0.13	0.73	2.11	0.18

**Fig 2:**
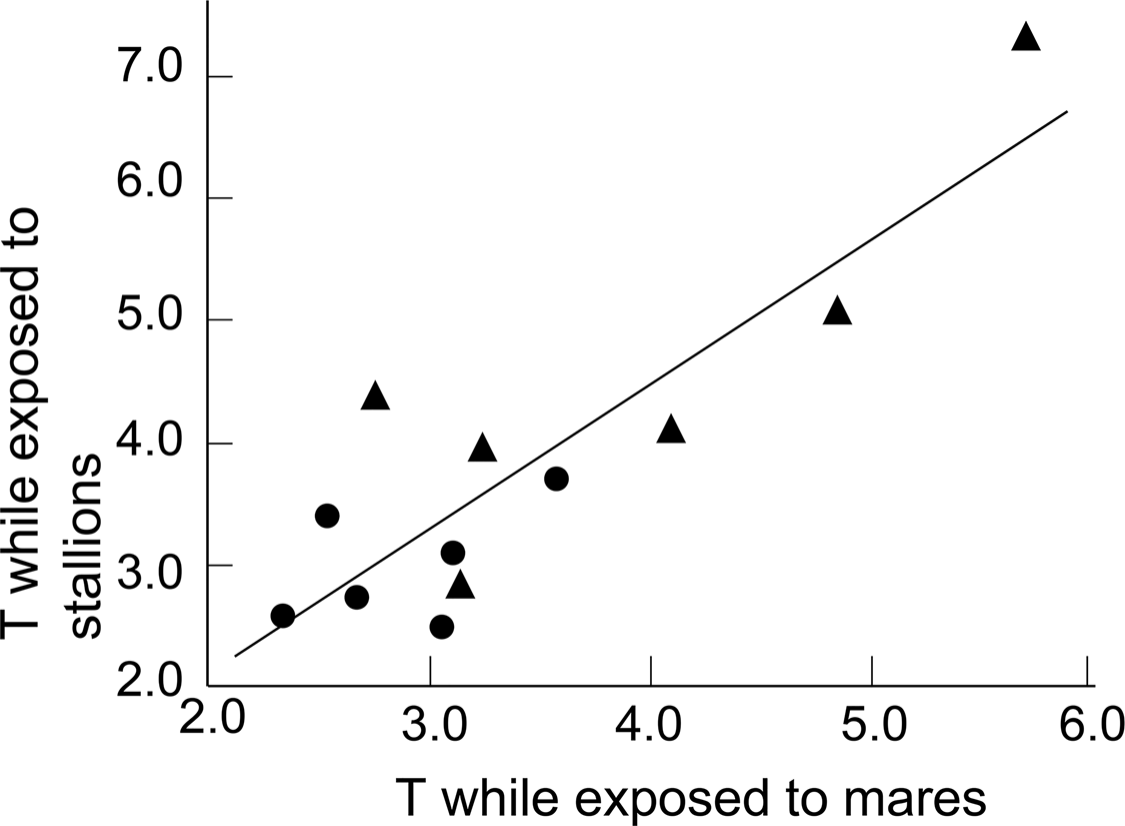
Testosterone levels. Correlation between average peripheral plasma testosterone levels (“T”; in nmol/L) while exposed to mares or to stallions (Kendalls’ tau = 0.58, p = 0.009). Stallions were either first exposed to stallions and then to mares (triangles) or vice versa (circles). The line gives the regression.

Average peripheral plasma testosterone levels were correlated across treatments, i.e. some stallions had consistently higher testosterone levels than others (Fig 2). Stallions that had first been exposed to other stallions showed on average higher mean testosterone levels than those that had first been exposed to mares (Fig 2; between-subject effect in Table 3). Sperm velocities were also correlated across treatments (VCL: tau = 0.49, p = 0.04; VSL: tau = 0.53, p = 0.02; VAP: tau = 0.45, p = 0.05), but sperm numbers were not (tau = 0.09, p = 0.70).

**Table 3:** MANOVA on average peripheral blood testosterone levels during exposure to stallions or to mares.

	F	d.f.	p
*Between-subjects*:			
Stallions or mares first (order)	6.03	1, 10	0.03
*Within-subjects*:			
After exposure to stallions or mares	3.18	1, 10	0.11
After exposure to stallions or mares x order	1.98	1, 10	0.19

**Fig 3:**
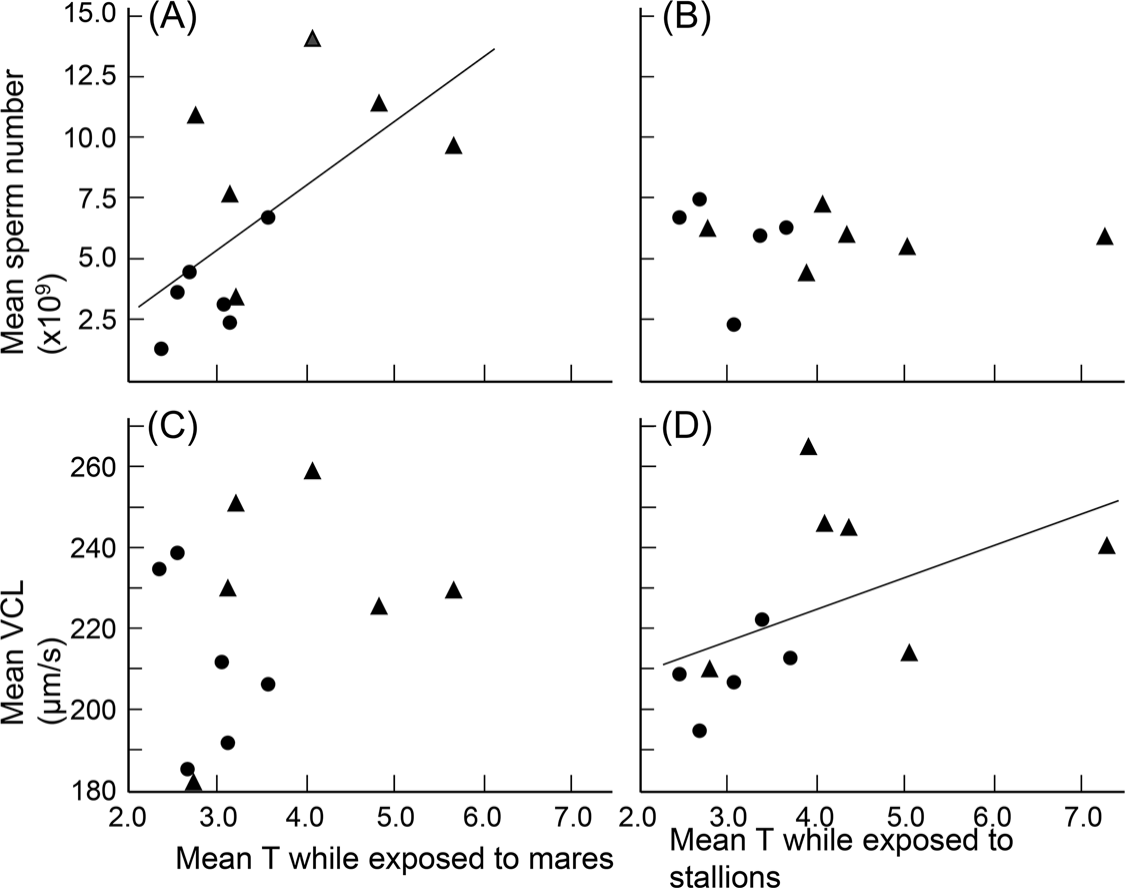
Semen characteristics versus testosterone levels. Semen characteristics versus mean peripheral plasma testosterone levels (“T”; in nmol/L). Mean sperm number (A) after exposure to mares versus T during exposure to mares (tau = 0.45, p = 0.04), and (B) after exposure to stallions against T during exposure to stallions (tau = -0.31, p = 0.19). (C) Mean VCL after exposure to mares against T during exposure to mares (tau = 0.18, p = 0.41), and (D) after exposure to stallions against T during exposure to stallions (tau = 0.49, p = 0.04). The analogous correlations between T and the other sperm velocity measures were never significant (|tau| always < 0.30, p always > 0.17). Stallions were either first exposed to stallions and then to mares (triangles) or vice versa (circles). The lines give the regressions to emphasize the direction of significant correlations.

Mean testosterone blood levels during the eight weeks of exposure to mares were useful predictors of mean sperm numbers in the corresponding ninth week (Fig 3A). No such correlation was found after exposure to stallions (Fig 3B). Among the sperm velocity measures, only VCL measured after exposure to stallions was correlated to testosterone blood levels during the corresponding exposure (Fig 3C and 3D).

## Discussion

Most sperm competition models assume that sperm compete in some kind of raffle [9]. Models that allow for facultative sperm allocation (e.g. [46]) therefore predict plastic responses in reaction to cues from the environment that help to assess risks of sperm competition. Here we compared four social situations that are likely to produce cues that may be extreme with respect to anticipated sperm competition: exposure to stallions before or after exposure to mares, and exposure to mares before or after exposure to stallions. We found strong effects of the order of the different types of exposures on ejaculate characteristics. Consequently, the kind of exposure (to several stallions or to one mare) mattered, too, even if their effects were statistically non-significant after controlling for order effects. Highest sperm numbers were found after exposure to mares, but only after previous exposure to other stallions, while sperm numbers were lowest after exposure to mares when stallions had not been exposed to other stallions yet. These observations correspond well with predictions from raffle-based models for facultative responses (see, for example, Table 4 in [9]).

We found VCL, one of our three measures of sperm velocity, to be affected by the order of our treatment: stallions that were first exposed to other stallions seemed to produce faster sperm than stallions that were exposed to mares before exposure to other stallions. There was even a positive correlation between VCL and mean testosterone levels during exposure to stallions (see below). However, the corresponding analyses on the other two measurements of sperm velocity were not significant, and we had no a priori expectancy of the relative importance of the different measures in the current context. Therefore, even if the three velocity measures we used here were to a high degree correlated, further studies are necessary to better understand the effects of social environments on sperm velocity.

We chose eight weeks per type of exposure and tested in the ninth week in order to cover a full cycle of spermatogenesis that comprises spermatocytogenesis, meiosis, and spermiogenesis [47]. Our study therefore includes possible effects of social cues on the very first stages of spermatogenesis. As we could only study ejaculates, it remains unclear whether and how sperm production is quantitatively affected by the treatment. However, shorter periods of exposure to social cues have been predicted to affect semen characteristics such as sperm velocity. Sperm velocity is determined by sperm morphology and ATP content [6] that may both mainly be determined during the two weeks of differentiation of spherical spermatids into mature spermatozoa in the horse [28]. The observed treatment effects on one sperm velocity measure therefore suggest that at least later stages of sperm production can be affected by the social environment. Further studies including, for example, daily sampling of semen, would be necessary to test this hypothesis.

We studied blood testosterone levels in reaction to the treatment because numerous studies have found links between this hormone and the behaviour males show towards other males or towards females [37]. In some species, highest testosterone levels were found in all-male groups [48]. However, McDonnell and Murray [49] found in semi-feral horses that harem stallions had significantly higher testosterone concentrations than bachelor stallions. In their study, harem and bachelor stallions were kept on the same pasture and in close proximity, i.e. interactions between harem and bachelor stallions were possible and may have influenced testosterone secretion. Observations by Khalil et al. [35,50] seem to confirm these findings. Aurich et al. [51] observed that breeding stallions who were mostly kept singly had lower testosterone levels than sexually inactive stallions. Together, these different findings suggest that testosterone concentrations are rather driven by the social context than by sexual activity. However, when we simulated a bachelor herd by stabling six stallions together for several weeks and without contact to mares, we found comparatively high testosterone levels that were not higher than those of stallions stabled alone with a mare. Our experimental situations may hence not sufficiently simulate natural bachelor or harem bands to create corresponding responses in testosterone levels.

Stallions appeared to consistently differ in their mean testosterone blood levels across treatments, i.e. some stallions tended to have higher testosterone blood levels than others. However, these rather robust between-subject differences do not exclude that (i) there could be treatment-specific within-subject changes in testosterone blood levels, and (ii) that testosterone blood levels correlate with different behavioral, morphological, or physiological traits dependent on the social environment. We found that blood testosterone levels were useful predictors of mean sperm number after exposure to mares but not after exposure to stallions. This suggests that testosterone levels during exposure to a mare can be a proxy for a stallion’s mate preference and his willingness to invest into costly semen production [8,52]. Indeed, we [38] found that male testosterone blood levels changed in reaction to major histocompatibility (MHC) types of the mares the stallions were exposed to. In this parallel study with the same animals as used here (see Methods), the stallions showed higher testosterone levels and hence appeared to invest more into attractiveness and mate guarding when exposed to MHC-dissimilar mares than when exposed to MHC-similar mares. It would be interesting to see if these changes in testosterone levels are also linked to other male characteristics such as dominance behavior.

In conclusion, as predicted from sperm competition theory, stallions are able to adjust ejaculate characteristics to the social environment they experience. Blood testosterone can be a useful predictor of ejaculate characteristics under some circumstances, but probably not under others.
